# The formation of self‐concept and intrinsic value in arts‐related domains: Extending the generalized internal/external frame of reference model to music and visual arts

**DOI:** 10.1111/bjep.70038

**Published:** 2025-09-24

**Authors:** A. Katrin Arens, Daniel Fiedler, Johannes Hasselhorn, Jens Möller

**Affiliations:** ^1^ Department on Research on Education and Human Development and IDeA Research Center for Individual Development and Adaptive Education of Children at Risk DIPF | Leibniz Institute for Research and Information in Education Frankfurt am Main Germany; ^2^ Chair of Music Education Research Department of Didactics Friedrich‐Alexander‐Universität Erlangen‐Nürnberg Nürnberg Germany; ^3^ Institute of Psychology of Learning and Instruction Kiel University Kiel Germany

**Keywords:** academic achievement, academic self‐concept, I/E model, intrinsic value, music, visual arts

## Abstract

**Background:**

The internal/external frame of reference (I/E) model explains the formation of domain‐specific academic self‐concepts. The generalized I/E (GI/E) model extends the I/E model to other motivational constructs, such as intrinsic value, and to various school subjects. When extending the I/E model to various school subjects, the domains of music and visual arts have seldom been examined.

**Aims:**

This study examined a GI/E model that included achievements, self‐concepts and intrinsic values in eight school subjects, including music and visual arts.

**Sample:**

We used a sample of *N* = 442 German secondary school students.

**Methods:**

The analyses were built upon structural equation modelling.

**Results:**

The findings demonstrated negative achievement–self‐concept and achievement–intrinsic value relations between math‐like and verbal‐like domains, indicating contrast effects. In addition, the findings demonstrated positive achievement–self‐concept and achievement–intrinsic value relations among math‐like domains, indicating assimilation effects. The findings further indicated negative relations between physics achievement and music self‐concept, implying a contrast effect. In addition, the findings showed a positive relation between music achievement and English self‐concept and between visual arts achievement and German intrinsic value, implying assimilation effects. Academic self‐concept was found to fully mediate the relation between achievement and intrinsic value within the same domains and across different domains in most cases. The findings were shown to be invariant across gender groups.

**Conclusion:**

The present study adds to the broad field of research on the I/E and GI/E models by considering the two neglected domains of music and visual arts.

## INTRODUCTION

The internal/external frame of reference (I/E) model is a well‐examined framework to explain the formation of students' domain‐specific academic self‐concept (Marsh, 1986). The I/E model initially only included math and verbal self‐concepts, but it has also been extended to self‐concepts in various school subjects (e.g., Arens et al., 2018; Jansen et al., 2015; Marsh, Lüdtke, et al., 2015; Möller et al., 2006). However, arts‐related school subjects such as music or visual arts have seldom been examined in the I/E model (but see Lohbeck, 2023). In this study, we examined an I/E model extended to music and visual arts self‐concepts while studying a broad range of school subjects. Beyond academic self‐concept, we included intrinsic value as another motivational construct. We tested gender invariance to examine the generalizability of our findings.

### Academic self‐concept and the I/E model

Academic self‐concept is defined as students' self‐perceptions of competence. It is a core construct in educational psychology as it has substantial relations to academic achievement, motivation and educational choices (Trautwein & Möller, 2016). In the classic self‐concept model proposed by Shavelson et al. (1976), academic self‐concept has been assumed to be an overarching factor subsuming math and verbal self‐concepts. However, math and verbal self‐concepts have been found to be only slightly correlated so that they cannot be subsumed into an overarching factor of academic self‐concept (Marsh, 1986).

The I/E model has been formulated to explain the low correlation between math and verbal self‐concepts and offers a well‐examined model for the formation of math and verbal self‐concepts (Marsh, 1986). The I/E model assumes that two kinds of comparison processes are at play in the formation of domain‐specific academic self‐concept: First, using social comparisons, students compare their own achievement in one domain with other students' achievements in the same domain; second, using dimensional comparisons, students compare their own achievement in one domain (e.g., math achievement) with their own achievement in other domains (e.g., verbal achievement). The interplay of these two comparison processes is assumed to lead to an idiosyncratic pattern of achievement–self‐concept relations: Within domains, achievement and self‐concept are positively related (math achievement and math self‐concept; verbal achievement and verbal self‐concept). These positive within‐domain relations are assumed to be due to social comparisons – relatively high achievement in one domain fosters students' self‐concept in the same domain. Across domains, achievement and self‐concept are negatively related (math achievement and verbal self‐concept; verbal achievement and math self‐concept). These negative cross‐domain relations are assumed to be due to dimensional comparisons – relatively high achievement in one domain weakens the self‐concept in another contrasting domain. This is known as a contrast effect. The interplay of the two comparison processes is assumed to lead to a positive relation between math and verbal achievements but to a near‐zero and relatively smaller correlation between math and verbal self‐concepts. These assumptions of the I/E model have been empirically validated by numerous studies, including meta‐analyses (Möller et al., 2009, 2020), experimental studies (Möller & Köller, 2001), longitudinal studies (Möller et al., 2011), intervention studies (Hörsch et al., 2025), and diary studies (Möller & Husemann, 2006).

### Extension to intrinsic value

The generalized internal/external frame of reference (GI/E) model (Möller et al., 2016; Möller & Marsh, 2013) extends the original I/E model to other motivational constructs beyond self‐concept. For instance, intrinsic value – defined as students' liking and enjoyment of a subject – has been found to show a similar pattern of achievement relations, i.e., positive within‐domain relations and negative cross‐domain relations (e.g., Arens & Preckel, 2018; Gaspard et al., 2018; Goetz et al., 2008; Schurtz et al., 2014). Hence, social and dimensional comparisons are also involved in the formation of intrinsic value. Academic self‐concept has been found to act as a mediator in the within‐domain and cross‐domain relations between achievement and intrinsic value (Gaspard et al., 2018; Goetz et al., 2008; Guo et al., 2017; Schurtz et al., 2014; van der Westhuizen et al., 2023). This finding corresponds well to the assumption of expectancy–value theory (EVT) according to which students start liking those domains in which they think they are good at (Wigfield & Eccles, 2000). This assumption also fits well with Self‐Determination Theory (SDT), according to which self‐perceptions of competence are a prerequisite for the formation of intrinsic motivation (Deci & Ryan, 2000). In fact, previous academic self‐concept has been found to be related to later intrinsic value (Arens et al., 2019; Lauermann et al., 2017; Marsh et al., 2005).

### Extension to various school subjects

Given the low correlation between math and verbal self‐concepts, the Shavelson model of academic self‐concept has been revised to the Marsh/Shavelson model of academic self‐concept (Marsh, 1990). The Marsh/Shavelson model assumes that students display separate academic self‐concepts for all school subjects including core and non‐core school subjects. These domain‐specific academic self‐concepts can be located on a continuum ranging from math‐like to verbal‐like academic self‐concepts. Two higher‐order factors (a higher‐order math self‐concept and a higher‐order verbal self‐concept) are superordinate to the domain‐specific academic self‐concepts. First‐order math‐like self‐concepts (e.g., physics self‐concept) have higher loadings on the higher‐order math self‐concept and first‐order verbal‐like self‐concepts (e.g., foreign language self‐concept) have higher loadings on the higher‐order verbal self‐concept.

The original I/E model only includes math and verbal achievements and math and verbal self‐concepts. The GI/E model not only extends the original I/E model to other motivational constructs (see above) but also to other school subjects (for an overview, see Möller, 2024). When including various school subjects in a GI/E model, negative achievement–self‐concept relations were found between domains located at the opposite ends of the math–verbal continuum of the Marsh/Shavelson model, such as math and verbal domains. This reflects the contrast effect of the original I/E model. Positive achievement–self‐concept relations were found between domains located close to each other at the math end of the math–verbal continuum, such as math and physics (Jansen et al., 2015; Marsh, Lüdtke, et al., 2015; Möller et al., 2006). Positive cross‐domain achievement–self‐concept relations are denominated as assimilation effects and depict that, contrary to contrast effects, higher achievement in one domain (math) fosters the self‐concept in a related domain (physics). Different language domains, such as the language of instruction and the first foreign language, present two domains, both located at the verbal end of the math–verbal continuum of the Marsh/Shavelson model. However, achievement–self‐concept relations across two verbal domains were found to be negative or non‐significant, implying rather a contrast effect than an assimilation effect between languages (Möller et al., 2020).

### Arts‐related self‐concepts and the I/E model

The I/E and GI/E models have been studied for academic self‐concepts and intrinsic values related to a variety of core (e.g., math, language of instruction, first foreign language) and non‐core school subjects (e.g., history, biology; see e.g., Arens et al., 2018; Jansen et al., 2015; Marsh, Lüdtke, et al., 2015; Möller et al., 2006). However, the school curriculum also includes arts‐related school domains such as music and visual arts. Music self‐concept is defined as students' self‐perceptions regarding musical abilities (e.g., Vispoel, 2003). Music self‐concept has been found to be related to motivation to engage in music (e.g., Fiedler & Spychiger, 2017; Schnare et al., 2012; West, 2013) and to achievement in music including school grades and musical competencies (e.g., Demorest et al., 2017; Fiedler et al., 2024; Lill et al., 2020; Morin et al., 2016). Little research has been done regarding students' self‐concept in visual arts. In previous studies, items related to visual arts self‐concept and items related to other arts‐related self‐concepts (Vispoel, 1995: dance, dramatic arts, visual arts and music skills self‐concepts; Mansour et al., 2018: dance, drama, film/video making, music and visual arts self‐concepts; Yeung et al., 2000, 2001: music, visual arts, dance and drama self‐concepts) were pooled to a common scale of arts self‐concept. Alternatively, the separate arts‐related self‐concept factors were integrated into a higher‐order factor of arts self‐concept. However, visual arts self‐concept only showed moderate to low correlations to other arts‐related self‐concepts (e.g., music, dance and drama self‐concepts) (*r* = .106 to .139; Marsh & Roche, 1996). This argues for a separate consideration of visual arts self‐concept.

In this study, we included music and visual arts self‐concepts in the GI/E model. Arts‐related school subjects such as music and visual arts are distinct from other school subjects as they share a mixed school‐based and non‐school‐based character; music and visual arts are taught at school and have a curriculum, but they are also often part of students' leisure time activities and implemented outside school. Hence, music and visual arts are taught in primary and secondary education schools as regular school subjects. Still, some students also take additional private extracurricular lessons or attend courses outside of school to learn and practice music and visual arts.

A major question concerns the existence of contrast and assimilation effects when incorporating arts‐related school subjects into a GI/E model along with other school subjects. Some reasons argue for an assimilation between music and visual arts. Vispoel (1995) proposed that arts‐related self‐concepts (i.e., dance, dramatic arts, visual arts and music skills self‐concepts) constitute a separate higher‐order factor that is distinct from the higher‐order academic and non‐academic self‐concept factors. Thus, an assimilation might exist between music and visual arts as they belong to the same higher‐order construct. No relations would be assumed to other academic domains as they are separately represented in another higher‐order factor. In addition, as described above, music and visual arts are both characterized by a mixed school‐based and non‐school‐based character. This shared character might also provoke an assimilation between music and visual arts as well as a contrast effect between music and visual arts on the one hand and other domains on the other hand that display a pure school‐based character.

Other studies, however, have provided empirical evidence that music self‐concept is related to other academic domains. In this context, we examine whether music and visual arts self‐concepts are more math‐like or more verbal‐like on the math–verbal continuum of the Marsh/Shavelson model (Marsh, 1990). Previous studies showed positive relations between music self‐concept, school grades in music, and academic self‐concepts addressing verbal subjects. However, the relations between music self‐concept, school grades in music, and academic self‐concepts addressing math subjects were relatively small (Arens et al., 2022; Morin et al., 2016). Based on this, an assimilation effect might be expected between music and verbal domains, but no or even a contrast effect might be expected between music and math.

Lohbeck (2023) presented a first study that included music self‐concept, intrinsic value, and achievement in a GI/E study beyond math and German self‐concepts, intrinsic values, and achievements. The findings showed positive achievement–self‐concept and achievement–intrinsic value relations within the domain of music. Across domains, German achievement was positively related to music intrinsic value, and music achievement was positively related to German intrinsic value, reflecting the presumptive assimilation effect between music and verbal domains. Math achievement was negatively related to music intrinsic value, reflecting a contrast effect between music and math. In further analyses, Lohbeck showed that self‐concept served as a mediator in the relations between achievement and intrinsic value, replicating the findings from previous studies (see above).

### Gender invariance

As a test for generalizability, a variety of studies have examined whether the findings on the GI/E and I/E models depend on student characteristics such as gender (Arens & Preckel, 2018; van der Westhuizen et al., 2023; for a meta‐analysis, see Möller et al., 2009). The original I/E model and the GI/E model extended to various school subjects and intrinsic value have been found to be invariant across gender groups. Therefore, the relations among achievement, self‐concept, and intrinsic value did not differ between boys and girls, indicating similar contrast and assimilation effects. These findings support the generalizability of the I/E and GI/E models.

### The present study

In this study, we used a sample of German students to examine a GI/E model that includes various domains (math, German, English, physics, biology, and second foreign language) and two arts‐related domains, i.e., music and visual arts. This allowed us to investigate possible contrast and assimilation effects among music, visual arts, and various other school subjects. Beyond academic self‐concept, we included intrinsic value related to these subjects as another motivational construct. Therefore, we could test the GI/E model with intrinsic value as an outcome variable. In addition, we could test whether academic self‐concept mediated the relation between achievement and intrinsic value. We further examined the invariance of our GI/E model across gender groups to test its generalizability.

To account for the mixed school‐based and non‐school‐based character, self‐concepts and intrinsic values in music and visual arts were measured explicitly with regard to music and visual arts as school subjects. To this aim, we adapted established academic self‐concept and intrinsic value scales to the domains of music and visual arts (see below). We asked students for their self‐perceptions in music and visual arts as school subjects.^1^ We therefore attempted to integrate music and visual arts self‐concepts and intrinsic values into the range of academic motivational variables to extend the GI/E model.

## METHOD

### Sample

The study was approved by the Ministry of General Education and Vocational Training, Science, Research and Culture of Schleswig‐Holstein. Data collection took place in 2019 and was administered by trained research assistants. The participating students and their parents provided written informed consent to participate. Participation was voluntary, and all data provided were treated confidentially and anonymously.

We used a sample of *N* = 442 German students (*n* = 180 male, *n* = 256 female, *n* = 6 no gender indicated). The mean age of the student sample was *M* = 13.22 years (*SD* = 0.955) ranging from 11 to 17 years. The students attended Grades 7 to 9 (Grade 7: *n* = 154, Grade 8: *n* = 149, Grade 9: *n* = 139) of secondary school.^2^ The secondary school system in Germany consists of different ability tracks, including the academic, intermediate, vocational, and comprehensive tracks. The students considered in this study all attended the academic track that prepares students to enter university after 12 or 13 years of schooling (depending on the German federal state). The students came from two different schools both located in the German federal state of Schleswig‐Holstein. The majority of the participating students (*n* = 421) were born in Germany; *n* = 17 students were not born in Germany; for *n* = 4 students, no respective information was available. As an indicator of socioeconomic status, we asked the students to estimate the number of books available in their households (Paulus, 2009). The responses implied that most students came from households with a high level of socioeconomic status as *n* = 197 students (44.6%) indicated that more than 200 books are available, and *n* = 121 (27.4%) indicated that there are enough books to fit onto three shelves.^3^

### Measures

#### Academic self‐concept

Students' academic self‐concept was measured in eight domains, namely in math, German (as the native language for most students and language of instruction for all students), English (the first foreign language learned at school), second foreign language (*n* = 210 students learned French, *n* = 149 students learned Latin, *n* = 73 students learned Spanish as their second foreign language, and *n* = 10 students learned another second foreign language or had missing values on this variable), physics, biology, music, and visual arts. Each of the academic self‐concept scales consisted of three items. The items originate from the large‐scale longitudinal study ‘Bildungsprozesse, Kompetenzentwicklung und Selektionsentscheidungen im Vorschul‐ und Schulalter [Educational Processes, Development of Competences and Formation of Decision on Selection in Early and Primary Education]’ (BiKS; Weinert et al., 2013) and were adapted to the specific school subjects considered. The items were worded in parallel across the domains: ‘[Domain] is easy for me.’; ‘I learn new things quickly in [domain].’; ‘I am good at [domain].’ The students were asked to respond to the items using a 5‐point Likert scale indicating the extent to which the items apply to themselves (not at all – little – somewhat – fairly – strongly). The reliability estimates were good for all academic self‐concept scales both when considering Cronbach's Alpha (*α*) and McDonald's Omega (*ω*) (McNeish, 2018; Table 1).

**TABLE 1:** Reliability estimates of the academic self‐concept and intrinsic value scales.

	*α*	*ω*
Math self‐concept	.944	.945
German self‐concept	.886	.885
English self‐concept	.916	.916
Physics self‐concept	.926	.927
Biology self‐concept	.889	.890
Second foreign language self‐concept	.952	.950
Art self‐concept	.918	.920
Music self‐concept	.898	.898
Math intrinsic value	.867	.869
German intrinsic value	.808	.804
English intrinsic value	.856	.876
Physics intrinsic value	.870	.875
Biology intrinsic value	.830	.832
Second foreign language intrinsic value	.841	.862
Art intrinsic value	.907	.923
Music intrinsic value	.884	.892

Abbreviations: *α*, Cronbach's Alpha; *ω*, McDonald's Omega.

#### Intrinsic value

Students' intrinsic value was measured in the same eight domains as students' academic self‐concept. The respective scale consisted of two items worded in parallel across the domains: ‘How much do you look forward to lessons in [domain]?’; ‘How much would you like to have more lessons in [domain]?’. These items were also retrieved from the BiKS study. To answer the items, the same 5‐point Likert scale was applied as used for the academic self‐concept scales. The reliability estimates for the intrinsic value scales were good both when considering *α* and *ω* (Table 1).

#### Achievement

Students' achievement was measured using their self‐reported school grades in the eight domains considered. In Germany, school grades range from 1 to 6, with 1 depicting the best and 6 depicting the poorest. The grades were reversely coded for the analyses so that higher values indicated higher achievements. Previous studies provided evidence of validity for student‐reported school grades (Sticca et al., 2017).

### Statistical analyses

All analyses were built on structural equation modelling (Kline, 2005) and were conducted with Mplus 8.10 (Muthén & Muthén, 1998–2023). The domain‐specific academic self‐concept factors were defined by the three corresponding items, and the domain‐specific intrinsic value factors were defined by the two corresponding items. We included correlated uniquenesses between parallel‐worded items for both the academic self‐concept and the intrinsic value factors (Marsh & Hau, 1996). This corrects for the potential shared method variance due to the parallel wordings of the items. The school grades served as single‐item indicators for defining the achievement factors. In all analyses, we used the robust maximum likelihood estimator (MLR), which is robust against violations of the normal distribution (Hox et al., 2010). In addition, we used the option ‘type = complex’ to consider the hierarchical nature of the data as students were nested in classes (Stapleton, 2006). Missing values were treated using the full information maximum likelihood estimator (FIML) implemented in Mplus (Enders, 2010). The amount of missing data was small, ranging from 0.2% to 1.4% for the academic self‐concept measures, from 0.2% to 1.1% for the intrinsic value measures, and from 0.2% to 2.7% for the achievement measures.

The first model was a confirmatory factor analysis (CFA) that included all factors (academic self‐concept, intrinsic value, and achievement factors in the eight domains considered) defined by the respective items. The second model was the GI/E model in which the academic self‐concept and intrinsic value factors were regressed on the achievement factors (Figure S1 in the Online Supplements). The subsequent model was a mediation model in which we tested whether the within‐domain and cross‐domain relations between achievement and intrinsic value were mediated through academic self‐concept (Figure S2 in the Online Supplements). For this purpose, we applied the ‘model indirect’ option implemented in Mplus using the domain‐specific achievement measures as the independent variables, the domain‐specific academic self‐concepts as the mediator variables, and the domain‐specific intrinsic values as the dependent variables. In the following models, we tested the invariance of our GI/E model across gender groups (Millsap, 2011). To this aim, we stated a model of configural invariance assuming the same number and pattern of factors for boys and girls. In a subsequent model of loading invariance, the factor loadings were restricted to be of the same size for boys and girls. The invariance of factor loadings is needed to examine the invariance of factor variances and covariances. This was done in the next model which was used to examine whether the within‐domain and cross‐domain relations between achievement, academic self‐concept, and intrinsic value, as stated in the GI/E model, were the same across gender groups.

For model fit evaluation, we considered the comparative fit index (CFI) and the Tucker‐Lewis index (TLI), which both should be higher than .90 for an adequate model fit or higher than .95 for a good model fit (Hu & Bentler, 1999). We also considered the root mean square error of approximation (RMSEA) with values below .05 indicating a close fit and values between .05 and .08 representing a reasonable fit (Browne & Cudeck, 1993). We further inspected the standardized root mean square residual (SRMR) with values below .08 indicating a good model fit (Hu & Bentler, 1999). Measurement invariance can be stated as long as the change in the CFI did not drop by more than −.01 between two models with more and fewer invariance constraints (Cheung & Rensvold, 2002).

## RESULTS

The CFA model, including eight academic self‐concept factors, eight intrinsic value factors, and eight achievement factors (Model 1), had a good model fit (Table 2). The factors were well defined, as the items had substantial positive loadings on their corresponding factors (Table S1 of the Online Supplements). The factor correlations (Table S2 of the Online Supplements) indicated the separateness of all domain‐specific self‐concept and intrinsic value factors, including music and visual arts. Means and standard deviations are shown in Table S3 of the Online Supplements.

**TABLE 2:** Goodness‐of‐fit indices.

		*χ* ^2^	*df*	CFI	TLI	RMSEA	SRMR
Model 1	CFA model with eight self‐concept factors, eight intrinsic value factors, and eight achievement factors	877.935	676	.987	.978	.026	.023
Model 2	GI/E model	877.935	676	.987	.978	.026	.023
Model 3	GI/E mediation model	1235.537	760	.968	.953	.038	.068
Model 4	Gender invariance: Configural invariance	1845.550	1352	.968	.947	.041	.032
Model 5	Gender invariance: Loading invariance	1869.389	1376	.968	.948	.041	.034
Model 6	Gender invariance: Invariance of factor variances and covariances	2273.617	1678	.962	.949	.040	.077

*Note*: All *χ* ^2^ values are statistically significant (*p* < .001).

Abbreviations: CFA, confirmatory factor analysis; CFI, comparative fit index; RMSEA, root mean square error of approximation; SRMR, standardized root mean squared residual; TLI, Tucker‐Lewis index.

### The GI/E model

The GI/E model was statistically equivalent to the CFA model as the correlations among factors were replaced by the path coefficients for the relations among achievement, academic self‐concept, and intrinsic value factors (Table 2). According to the GI/E model assumptions, achievement and self‐concept were positively related within all domains (Table 3; for the factor correlations, see Tables S4 and S5 of the Online Supplements). Across domains, the findings indicated contrast effects between math‐like and verbal‐like domains given significant negative relations between German achievement and math self‐concept, German achievement and physics self‐concept, math achievement and German self‐concept, math achievement and English self‐concept, English achievement and math self‐concept, and physics achievement and second foreign language self‐concept. The results also showed a negative relation between biology achievement and second foreign language self‐concept indicating a contrast effect. Moreover, the results showed a positive relation between math achievement and physics self‐concept implying an assimilation effect between two math‐like domains. Regarding the arts‐related self‐concepts, we found a negative relation between physics achievement and music self‐concept implying a contrast effect. Moreover, we found a positive relation between music achievement and English self‐concept implying an assimilation effect.

**TABLE 3:** Standardized path coefficients of the generalized internal/external frame of reference (GI/E) model (Model 2).

	M_SC	G_SC	E_SC	P_SC	B_SC	FL_SC	VA_SC	Mu_SC	M_IV	G_IV	E_IV	P_IV	B_IV	FL_IV	VA_IV	Mu_IV
M_Ach on	.757*	−.116*	−.122*	.225*	−.049	.063	−.115	.061	.543*	−.153*	−.065	.209*	−.016	.048	−.065	.060
G_Ach on	−.151*	.693*	.011	−.204*	.030	−.074	−.003	−.009	−.047	.487*	−.060	−.181*	−.048	−.020	.006	.005
E_Ach on	−.136*	.034	.763*	−.027	−.057	.105	−.056	.051	−.135	.000	.729*	−.054	−.070	.101	−.082	−.028
P_Ach on	.056	−.097	−.097	.541*	.073	−.156*	−.025	−.114*	.039	−.111	−.121*	.445*	.123	−.170*	−.056	−.099
B_Ach on	.042	.021	−.055	.062	.453*	−.131*	−.058	−.031	−.044	.070	−.052	.030	.294*	−.088	−.060	−.029
FL_Ach on	.043	−.016	−.074	−.080	.001	.689*	.008	.060	.038	−.006	−.108	−.078	.017	.483*	−.031	.018
VA_Ach on	−.088	.000	−.085	−.035	.013	.037	.612*	−.031	−.066	.113*	−.022	.012	.083	.091	.553*	.006
Mu_Ach on	−.018	.026	.090*	−.037	.063	.062	.043	.536*	−.002	.042	.013	−.095	−.036	.023	.056	.408*

Abbreviations: B_Ach, biology achievement; B_IV, biology intrinsic value; B_SC, biology self‐concept; E_Ach, English achievement; E_IV, English intrinsic value; E_SC, English self‐concept; FL_Ach, second foreign language achievement; FL_IV, second foreign language intrinsic value; FL_SC, second foreign language self‐concept; G_Ach, German achievement; G_IV, German intrinsic value; G_SC, German self‐concept; M_Ach, math achievement; M_IV, math intrinsic value; M_SC, math self‐concept; Mu_Ach, music achievement; MU_IV, music intrinsic value; Mu_SC, music self‐concept; P_Ach, physics achievement; P_IV, physics intrinsic value; P_SC, physics self‐concept; VA_Ach, visual arts achievement; VA_IV, visual arts intrinsic value; VA_SC, visual arts self‐concept.

* *p* < .05.

The pattern of relations between achievement and intrinsic value was similar to the pattern of relations between achievement and academic self‐concept, although the relations were smaller in size. The within‐domain relations between achievement and intrinsic value were all positive and significant. Across domains, there were few negative relations between math‐like and verbal‐like domains, such as between math achievement and German intrinsic value, German achievement and physics intrinsic value, physics achievement and second language intrinsic value, and physics achievement and English intrinsic value, indicating contrast effects. Math achievement was positively related to physics intrinsic value, indicating an assimilation effect. Regarding the arts‐related self‐concepts, we found a positive relation between visual arts achievement and German intrinsic value. All other cross‐domain relations involving music and visual arts intrinsic values were non‐significant.

### Mediation through academic self‐concept

To test the assumption that academic self‐concept mediates the relations between achievement and intrinsic value within and across domains, we stated Model 3 (Table 2). To reduce model complexity, we only included mediation paths for the relations between achievement and intrinsic value that were found to be significant in Model 2. Within domains, the indirect effects were all significant and positive, indicating that domain‐specific achievement was positively related to the matching domain‐specific academic self‐concept that was itself positively related to the matching domain‐specific intrinsic value. With two exceptions (i.e., the relation between English achievement and English intrinsic value mediated through English self‐concept, and the relation between second foreign language achievement and second foreign language intrinsic value mediated through second foreign language self‐concept), all within‐domain relations were fully mediated through academic self‐concept as the direct relations became non‐significant (Table 4).

**TABLE 4:** Standardized effects from the mediated generalized internal/external frame of reference (GI/E) model (Model 3).

	Total effect	Indirect effect	Direct effect
Math achievement → math self‐concept → math intrinsic value	.545*	.675*	−.130
German achievement → German self‐concept → German intrinsic value	.486*	.507*	−.021
English achievement → English self‐concept → English intrinsic value	.730*	.569*	.161*
Physics achievement → physics self‐concept → physics intrinsic value	.451*	.479*	−.027
Biology achievement → biology self‐concept → biology intrinsic value	.295*	.376*	−.081
Second foreign language achievement → second foreign language self‐concept → second foreign language intrinsic value	.491*	.629*	−.138*
Visual art achievement → visual art self‐concept → visual art intrinsic value	.555*	.505*	.050
Music achievement → music self‐concept → music intrinsic value	.411*	.424*	−.013
Math achievement → German self‐concept → German intrinsic value	−.150*	−.084*	−.066
Math achievement → physics self‐concept → physics intrinsic value	.212*	.199*	.013
German achievement → physics self‐concept → physics intrinsic value	−.187*	−.181*	−.005
Physics achievement → second foreign language self‐concept → second foreign language intrinsic value	−.172*	−.143*	−.030
Physics achievement → English self‐concept → English intrinsic value	−.122*	−.072	−.050
Visual art achievement → German self‐concept → German intrinsic value	.111*	.001	.111*

* *p* < .05.

Across domains, the indirect effects of three relations (i.e., between math achievement and German intrinsic value mediated through German self‐concept, between German achievement and physics intrinsic value mediated through physics self‐concept, and between physics achievement and second foreign language intrinsic value mediated through second foreign language self‐concept) were negative and significant, corresponding to the contrast effect between math‐like and verbal‐like domains. These three relations were fully mediated through self‐concept as the direct effect became non‐significant. The indirect effect for the relation between math achievement and physics intrinsic value mediated through physics self‐concept was positive and significant, corresponding to the assimilation effect among math‐like domains. This relation was also fully mediated through academic self‐concept as the direct effect was non‐significant. Regarding the relation between visual arts achievement and German intrinsic value mediated through German self‐concept, the direct effect was positive and significant, but the indirect effect was non‐significant. Regarding the relation between physics achievement and English intrinsic value mediated through English self‐concept, both the direct and indirect effects were not significant (for the full pattern of regression paths and factor correlations of the GI/E mediation model, see Tables S6–S8 of the Online Supplements).

### Gender invariance

The gender invariance tests consisted of a model assuming configural invariance (Model 4; Table 2), a model assuming factor loading invariance (Model 5), and a model assuming invariance of factor variances and covariances (Model 6).^4^ Across these models, the CFI remained the same or only declined marginally so that gender invariance could be confirmed. In other words, boys and girls were not found to differ regarding the within‐domain and cross‐domain relations among achievement, self‐concept, and intrinsic value, and the GI/E model pattern seems to apply similarly for both gender groups.

## DISCUSSION

### Summary of findings

This was the first study to extend the GI/E model to self‐concepts and intrinsic values in music and visual arts. The findings corresponded to the GI/E model assumptions: Within domains, there were consistently positive and significant relations between achievement and self‐concept, and between achievement and intrinsic value, indicating that higher achievement in one domain is associated with higher levels of self‐concept and intrinsic value in the same domain (Möller et al., 2009, 2020). These findings imply that social comparisons are at play in the formation of domain‐specific academic self‐concepts and intrinsic values. Our findings further imply that dimensional comparisons are at play in the formation of domain‐specific academic self‐concepts and intrinsic values. Considering the cross‐domain relations, our findings replicated contrast effects across math‐like and verbal‐like domains for both academic self‐concept and intrinsic value as outcome variables (Möller et al., 2009, 2020). The findings of our study further replicated the assimilation effect between math and physics as two math‐like domains (Jansen et al., 2015; Möller et al., 2009, 2020) as we found significant positive relations between math achievement and physics self‐concept, and between math achievement and physics intrinsic value.

Regarding music, the findings demonstrated a positive relation between music achievement and English self‐concept, and a negative relation between physics achievement and music self‐concept. These findings indicated an assimilation effect between music and verbal‐like domains and a contrast effect between music and math‐like domains. Regarding visual arts, our findings indicated an assimilation effect involving visual arts and verbal‐like domains, given the positive relation between visual arts achievement and German intrinsic value.

All within‐domain relations between achievement and intrinsic value were mediated through academic self‐concept, with most of the relations being fully mediated. We further tested whether the significant cross‐domain relations between achievement and intrinsic value were also mediated through academic self‐concept. We found evidence of this mediation with two exceptions: the relation between visual arts achievement and German intrinsic value was not found to be mediated through German self‐concept, and the relation between physics achievement and English intrinsic value was not found to be mediated through English self‐concept.

In invariance tests, we found evidence for gender invariance of our GI/E model. The within‐domain and cross‐domain relations between achievement, self‐concept, and intrinsic value related to eight domains were not found to vary across gender groups.

### Implications for theory and practice

As this study is the first study that includes self‐concepts and intrinsic values in music and visual arts in a GI/E model, there are many implications for theory and practice. The common practice of subsuming music and visual arts under an ‘artistic’ or ‘creative’ category should be critically reflected. In our study, music and visual arts self‐concepts and intrinsic values were found to form separate constructs that showed differential patterns of findings in the GI/E model. Hence, one cannot infer an ‘artistic’ person who has high levels of self‐concept and intrinsic value in both music and visual arts. Practitioners should instead consider music and visual arts as separate domains for which students form distinct self‐perceptions.

The findings of this study supported an assimilation effect between music and verbal‐like domains given a positive relation between music achievement and English self‐concept. This finding corresponds to the findings of other studies showing a close relation between music and verbal‐like domains including close relations between music and language‐related abilities (for a review, see Nayak et al., 2022; see also Ferreri & Verga, 2016; Taylor & Dewhurst, 2017; Turker & Reiterer, 2021). We found a negative relation between physics achievement and music self‐concept implying a contrast effect between music and math‐like domains. This finding corresponds to the findings of previous studies showing a separation between music and math domains (Fiedler et al., 2024; Morin et al., 2016; Vispoel, 2003). These findings counter the widespread assumption that music and math are closely associated domains or that music is positively related to achievement in all other domains (e.g., Schellenberg, 2004; Swaminathan & Schellenberg, 2019). The findings of the present study further supported an assimilation effect between visual arts and verbal‐like domains given a positive relation between visual arts achievement and German intrinsic value. Thoughts, emotions and imagination, which are all verbally represented, could be expressed in visual arts. Hence, both arts‐related domains examined here, i.e., music and visual arts, tend to show stronger associations with verbal domains than with math domains. This might also be traced back to the dual character of the arts‐related domains that is shared by verbal domains. The self‐concept and intrinsic value measures used in this study focused on the school‐based character of the domains. Still, music and visual arts have a dual character as they are part of the school curriculum but also part of students' leisure time and implemented outside school. This dual character also applies to the verbal domain because separate school subjects exist for the different languages, but verbal proficiency and skills are also implemented and used outside school. Still, the contrast and assimilation effects involving music and visual arts need further replication as they were only found for some math‐like (i.e., music and physics) and verbal‐like (i.e., music and English, visual arts, and German) domains in our study and did not generalize across all domain‐specific self‐concepts and intrinsic values as outcome variables.

The within‐domain and cross‐domain relations between achievement and intrinsic value were found to be mediated through academic self‐concept in most cases. Therefore, our findings support the assumptions of EVT and SDT according to which self‐perceptions of competence are a precursor for intrinsic value. In addition, this finding suggests that social and dimensional comparisons primarily address the formation of academic self‐concept rather than the formation of intrinsic value. This aligns with the definition of self‐concept that states that environmental experiences and their interpretation shape individuals' self‐concept (Shavelson et al., 1976); therefore, social and dimensional comparisons of achievement might form individuals' academic self‐concept that itself relates to intrinsic value. Accordingly, the relations between achievement and self‐concept are found to be usually higher than the relations between achievement and intrinsic value (Gaspard et al., 2018; van der Westhuizen et al., 2023). Interestingly, when testing whether the relation between visual arts achievement and German intrinsic value was mediated through German self‐concept, the indirect effect became non‐significant, whereas the direct effect was positive and significant. Hence, the assimilation effect between visual arts achievement and German intrinsic value was not found to be mediated through academic self‐concept. The finding implies a closer connection between achievement and motivation (e.g., intrinsic value) than between achievement and self‐concept in visual arts, whereas achievement and self‐concept are more closely related than achievement and motivation (e.g., intrinsic value) in other domains (Gaspard et al., 2018; van der Westhuizen et al., 2023). Further research is necessary to investigate the mechanisms underlying cross‐domain relations between achievement and intrinsic value involving arts‐related domains such as visual art.

The within‐domain and cross‐domain relations between achievement, self‐concept, and intrinsic value were found to be invariant across gender groups. Therefore, our study provided further evidence of the generalizability of the GI/E model across student characteristics when extending it by including music and visual arts (Arens & Preckel, 2018; Möller et al., 2009; van der Westhuizen et al., 2023). This finding indicates that boys and girls apply social and dimensional comparisons in a similar way for the formation of academic self‐concept and intrinsic value when considering a broad range of school subjects, including arts‐related school subjects such as music and visual arts.

### Limitations and directions for further research

The present study examined a GI/E model extended to self‐concepts and intrinsic values in music and visual arts. The main question addressed the occurrence of social and dimensional comparisons (including contrast and assimilation effects) when considering eight domains, including music and visual arts, by observing the within‐domain and cross‐domain relations between domain‐specific achievements, self‐concepts, and intrinsic values. Still, one has to bear in mind that the pattern of within‐domain and cross‐domain relations between domain‐specific achievements, self‐concepts, and intrinsic values only indicates but does not prove the implementation and application of social and dimensional comparisons. Hence, the observation of the pattern of within‐domain and cross‐domain relations does not allow insight into the psychological mechanisms underlying social and dimensional comparisons. To substantiate and explore the latter, experimental studies (Möller & Köller, 2001), longitudinal studies (Möller et al., 2011), intervention studies (Hörsch et al., 2025), and diary studies (Möller & Husemann, 2006) are needed (for an overview, see Möller, 2024). Wolff et al. (2020) replicated the I/E model assumptions using implicit and explicit self‐concepts. This finding shows that dimensional comparisons occur unconsciously, emphasizing the presence and impact of dimensional comparisons.

Previous studies examined potential moderator variables that might affect the strength of the negative relation between math (verbal) achievement and verbal (math) self‐concept displaying dimensional comparisons (Helm et al., 2016; Rost et al., 2005; Schmidt et al., 2018). The integrative study by Wolff et al. (2021) revealed that (1) students' belief in the negative interdependence of math and verbal abilities, and (2) students' belief that the subjects of math and German are dissimilar rather than similar operate as respective moderator variables. Further studies are needed to investigate the role of moderator variables in GI/E models, specifically I/E models extended to various domains including arts‐related domains and various motivational constructs such as intrinsic value.

We included achievements, self‐concepts, and intrinsic values in eight domains in our GI/E model. Further extensions of the GI/E model are still conceivable. Regarding domains, it would be interesting to integrate physical ability (see Arens & Preckel, 2018). Physical ability is similar to the music and visual arts domains as it also has a mixed school‐based and non‐school‐based character. In fact, physical ability is a school subject but also plays an important part in students' leisure time. A GI/E model including visual arts, music, and physical ability would help elucidate contrast and assimilation effects across a wide range of school subjects with different characteristics (i.e., core and non‐core school subjects and school subjects with a mixed school‐based and non‐school‐based character). Regarding motivational variables, beyond intrinsic value, the GI/E model has been expanded to text anxiety (Arens et al., 2017) and goal orientations (Dörendahl et al., 2021), and these extensions should be implemented when considering music and visual arts as further domains.

In this study, the self‐concept and intrinsic value measures specifically addressed music and visual arts as school subjects. However, music and visual arts are also often part of students' extracurricular activities. Hence, we cannot exclude that students also refer to non‐school or extracurricular experiences beyond school experiences (as requested) when responding to the respective items. Hence, future research should test the generalizability of our findings across measurement instruments that tap experiences with music and visual arts inside and outside school to different extents. Students with high levels of activity and expertise in outside‐school experiences in music and visual arts might differ from those with no or little exposure to music and visual arts outside school (e.g., Fiedler et al., 2024). Future research is thus necessary to test the generalizability of our findings across samples of students with varying levels of experience with music and visual arts inside and outside school.

We used gender as an important student characteristic to examine the generalizability of our GI/E model. Existing studies have demonstrated that the I/E and GI/E models are rather robust across other student characteristics such as secondary school type (Arens et al., 2018) or culture (Marsh, Abduljabbar, et al., 2015), but this should also be proven for a GI/E model including music and visual arts. The findings of several studies indicate that early elementary school students do not yet use dimensional comparisons for the formation of academic self‐concept and intrinsic value; the I/E and GI/E models can only can be fully supported in later elementary and secondary school (Lohbeck & Möller, 2017; van der Westhuizen et al., 2023; Weidinger et al., 2019; see also Wan et al., 2021). This finding on age/grade level effects regarding the use of dimensional comparisons should be replicated using our GI/E model extended to music and visual arts. As the present study suffers from a relatively small sample consisting of students from only two schools, a larger and more diverse sample should be realized in further replication studies.

Achievement was measured using student self‐reported grades. Although previous research supported the accuracy of student self‐reported grades (Sticca et al., 2017), there is a debate on the accuracy and thus reliability of this achievement measure (Kuncel et al., 2005). Therefore, alternative achievement indicators, including teacher‐reported grades and standardized achievement test scores, should be used to replicate the findings of the present study.

This study used cross‐sectional data that is not ideal for conducting mediation analyses as all variables (independent variable, mediator variable, and outcome variable) were measured at the same time. Therefore, we could not test the causal order implicitly assumed in mediation analyses (Mitchell & Maxwell, 2013). Additionally, due to the cross‐sectional nature of the data, the study could not examine relations across time. Hence, the present data set should be expanded with longitudinal data. This would enable a more sophisticated approach to testing the mediation model and applying further theoretical models. The reciprocal I/E model (RI/E model; Möller et al., 2011) extends the original I/E model to longitudinal relations; the generalized RI/E model (GRI/E model; Arens & Niepel, 2023) combines the RI/E model and the GI/E model and thus investigates longitudinal within‐domain and cross‐domain relations when considering different school subjects and motivational constructs. Therefore, the GRI/E model would provide a sophisticated framework to test longitudinal relations within and across various domains including music and visual arts.

## AUTHOR CONTRIBUTIONS

**A. Katrin Arens:** Conceptualization; investigation; writing – original draft; methodology. **Daniel Fiedler:** Writing – review and editing; data curation. **Johannes Hasselhorn:** Data curation; writing – review and editing. **Jens Möller:** Writing – review and editing.

## FUNDING INFORMATION

Preparation of this article was supported by a Heisenberg fellowship grant from the German Research Foundation to A.K. Arens (AR 877/3‐1).

## CONFLICT OF INTEREST STATEMENT

There is no conflict of interest associated with this manuscript.

## Supporting information


Appendix S1.

## Data Availability

Research data are not shared.

## Footnotes

^1^
There are other instruments ‐ for instance, the Music Self‐Perception Inventory (MUSPI, e.g., Fiedler et al., 2024; Morin et al., 2016) ‐ that assess music self‐concept on a more general level, that is, not specially related to the corresponding school subject but to music as a life domain.

^2^
The data used here were already used in two studies addressing music self‐concept (Arens et al., 2022; Fiedler et al., 2024), but none of the studies tested the GI/E model, including art and music self‐concepts.

^3^
The full range of response options is as follows: No or only very few books: *n* = 9 (2,0%); enough books to fit in a single shelf: *n* = 25 (5,7%); enough books to fit in shelves: *n* = 84 (19,0%); enough books to fit in three shelves: *n* = 121 (27,4%); more than 200 books: *n* = 197 (44,6%); no information available: *n* = 6 (1,4%).

^4^
The gender invariance tests were conducted based on a sample size of *N* = 436 (*n* = 180 boys, *n* = 256 girls), as six students had missing data on the gender variable.
