# Supplementary material for: The formation of self‐concept and intrinsic value in arts‐related domains: Extending the generalized internal/external frame of reference model to music and visual arts
Source: Br J Educ Psychol. 2025 Sep 24;96(2):558–76. doi: 10.1111/bjep.70038 (PMC13155071; doi:10.1111/bjep.70038)
Supplement: Supplementary file 1 — Appendix S1. [file BJEP-96-558-s001.docx]

**Online Supplements**

The Formation of Self-Concept and Intrinsic Value in Arts-Related Domains: Extending the Generalized Internal/External Frame of Reference Model to Music and Visual Arts

Table S1

*Standardized Factor Loadings from Model 1 of the Main Manuscript*

| Math self-concept |  |
| --- | --- |
| Item 1 | .942* |
| Item 2 | .885* |
| Item 3 | .940* |
| German self-concept |  |
| Item 1 | .899* |
| Item 2 | .814* |
| Item 3 | .835* |
| English self-concept |  |
| Item 1 | .884* |
| Item 2 | .872* |
| Item 3 | .900* |
| Physics self-concept |  |
| Item 1 | .903* |
| Item 2 | .893* |
| Item 3 | .901* |
| Biology self-concept |  |
| Item 1 | .875* |
| Item 2 | .817* |
| Item 3 | .880* |
| Second foreign language self-concept |  |
| Item 1 | .932* |
| Item 2 | .916* |
| Item 3 | .941* |
| Visual Arts self-concept |  |
| Item 1 | .913* |
| Item 2 | .880* |
| Item 3 | .884* |
| Music self-concept |  |
| Item 1 | .867* |
| Item 2 | .868* |
| Item 3 | .853* |
| Math intrinsic value |  |
| Item 1 | .839* |
| Item 2 | .928* |
| German intrinsic value |  |
| Item 1 | .811* |
| Item 2 | .823* |
| English intrinsic value |  |
| Item 1 | .846* |
| Item 2 | .899* |
| Physics intrinsic value |  |
| Item 1 | .870* |
| Item 2 | .891* |
| Biology intrinsic value |  |
| Item 1 | .756* |
| Item 2 | .949* |
| Second foreign intrinsic value |  |
| Item 1 | .844* |
| Item 2 | .872* |
| Visual Arts intrinsic value |  |
| Item 1 | .896* |
| Item 2 | .941* |
| Music intrinsic value |  |
| Item 1 | .884* |
| Item 2 | .906* |

*Note.* * *p* < .05.

Table S2

*Factor Correlations from Model 1 of the Main Manuscript*

|  | M_SC | G_SC | E_SC | P_SC | B_SC | FL_SC | VA_SC | Mu_SC | M_IV | G_IV | E_IV | P_IV | B_IV | FL_IV | VA_IV | Mu_IV | M_Ach | G_Ach | E_Ach | P_Ach | B_Ach | FL_Ach | VA_Ach |
| --- | --- | --- | --- | --- | --- | --- | --- | --- | --- | --- | --- | --- | --- | --- | --- | --- | --- | --- | --- | --- | --- | --- | --- |
| G_SC | .055 |  |  |  |  |  |  |  |  |  |  |  |  |  |  |  |  |  |  |  |  |  |  |
| E_SC | .049 | .335* |  |  |  |  |  |  |  |  |  |  |  |  |  |  |  |  |  |  |  |  |  |
| P_SC | .564* | .052 | .123* |  |  |  |  |  |  |  |  |  |  |  |  |  |  |  |  |  |  |  |  |
| B_SC | .169* | .359* | .148* | .327* |  |  |  |  |  |  |  |  |  |  |  |  |  |  |  |  |  |  |  |
| FL_SC | .232* | .319* | .320* | .208* | .236* |  |  |  |  |  |  |  |  |  |  |  |  |  |  |  |  |  |  |
| VA_SC | -.127* | .204* | .112 | -.006 | .223* | .093* |  |  |  |  |  |  |  |  |  |  |  |  |  |  |  |  |  |
| MU_SC | .165* | .328* | .252* | .069 | .166* | .236* | .279* |  |  |  |  |  |  |  |  |  |  |  |  |  |  |  |  |
| M_IV | .793* | .092* | .043 | .451* | .071 | .199* | -.084 | .196* |  |  |  |  |  |  |  |  |  |  |  |  |  |  |  |
| G_IV | -.028 | .726* | .210* | .042 | .263* | .249* | .268* | .241* | .095 |  |  |  |  |  |  |  |  |  |  |  |  |  |  |
| E_IV | .036 | .283* | .800* | .114* | .081 | .276* | .154* | .243* | .118* | .288* |  |  |  |  |  |  |  |  |  |  |  |  |  |
| P_IV | .438* | -.018 | .080 | .854* | .264* | .125* | .010 | .063 | .438* | .025 | .169* |  |  |  |  |  |  |  |  |  |  |  |  |
| B_IV | .108* | .157* | .056 | .278* | .771* | .162* | .160* | .088 | .169* | .281* | .138* | .338* |  |  |  |  |  |  |  |  |  |  |  |
| FL_IV | .197* | .205* | .256* | .130* | .186* | .834* | .119* | .171* | .243* | .301* | .264* | .168* | .238* |  |  |  |  |  |  |  |  |  |  |
| VA_IV | -.110 | .129* | .043 | .011 | .128* | .046 | .845* | .212* | -.105 | .223* | .114 | .036 | .117 | .116* |  |  |  |  |  |  |  |  |  |
| Mu_IV | .122* | .185* | .067 | .070 | .058 | .139* | .183* | .770* | .152* | .229* | .142* | .169* | .062 | .181* | .268* |  |  |  |  |  |  |  |  |
| M_Ach | .672* | .202* | .108* | .412* | .206* | .274* | -.051 | .192* | .471* | .088 | .071 | .313* | .144* | .195* | -.052 | .118* |  |  |  |  |  |  |  |
| G_Ach | .185* | .618* | .299* | .115* | .243* | .321* | .109* | .231* | .145* | .441* | .197* | .045 | .120* | .262* | .074 | .140* | .509* |  |  |  |  |  |  |
| E_Ach | .137* | .366* | .614* | .138* | .180* | .424* | .047 | .225* | .075 | .248* | .522* | .056 | .087 | .331* | -.002 | .100* | .432* | .602* |  |  |  |  |  |
| P_Ach | .398* | .200* | .114* | .544* | .278* | .149* | .018 | .104 | .267* | .107 | .055 | .416* | .220* | .086 | -.017 | .043 | .613* | .493* | .437* |  |  |  |  |
| B_Ach | .259* | .252* | .121* | .258* | .485* | .202* | .081 | .161* | .130* | .219* | .069 | .168* | .321* | .158* | .045 | .091 | .432* | .452* | .401* | .491* |  |  |  |
| FL_Ach | .251* | .327* | .289* | .134* | .241* | .642* | .040 | .246* | .166* | .231* | .198* | .057 | .143* | .471* | -.010 | .130* | .478* | .575* | .617* | .427* | .494* |  |  |
| VA_Ach | -.008 | .227* | .078* | .049 | .193* | .154* | .566* | .158* | -.017 | .270* | .076 | .038 | .165* | .172* | .499* | .126* | .184* | .360* | .276* | .248* | .337* | .238* |  |
| Mu_Ach | .132* | .264* | .213* | .081 | .224* | .268* | .178* | .537* | .094 | .226* | .117* | -.004 | .089 | .198* | .157* | .388* | .323* | .419* | .347* | .297* | .347* | .376* | .356* |

*Note.* M_SC = math self-concept; G_SC = German self-concept; E_SC = English self-concept; P_SC = physics self-concept; B_SC = biology self-concept; FL_SC = second foreign language self-concept; VA_SC = visual arts self-concept; Mu_SC = music self-concept; M_IV = math intrinsic value; G_IV = German intrinsic value; E_IV = English intrinsic value; P_IV = physics intrinsic value; B_IV = biology intrinsic value; FL_IV = foreign language intrinsic value; VA_IV = visual arts intrinsic value; MU_IV = music intrinsic value; M_Ach = math achievement; G_Ach = German achievement; E_Ach = English achievement; P_Ach = physics achievement; B_Ach = biology achievement; FL_Ach = foreign language achievement; VA_Ach = visual arts achievement; Mu_Ach = music achievement.

* *p* < .05.

Table S3

*Means and Standard Deviations*

|  | M_SC | G_SC | E_SC | P_SC | B_SC | FL_SC | VA_SC | Mu_SC |
| --- | --- | --- | --- | --- | --- | --- | --- | --- |
| *M* | 3.54 | 3.65 | 3.86 | 3.28 | 3.67 | 3.32 | 3.81 | 3.87 |
| *SD* | 1.00 | 0.75 | 0.80 | 0.94 | 0.72 | 1.03 | 1.00 | 0.84 |
|  | M_IV | G_IV | E_IV | P_IV | B_IV | FL_IV | VA_IV | Mu_IV |
| *M* | 3.11 | 3.04 | 3.37 | 2.73 | 2.87 | 2.61 | 3.37 | 3.32 |
| *SD* | 1.11 | 0.97 | 1.03 | 1.11 | 1.05 | 1.04 | 1.26 | 1.14 |
|  | M_Ach | G_Ach | E_Ach | P_Ach | B_Ach | FL_Ach | VA_Ach | Mu_Ach |
| *M* | 4.36 | 4.48 | 4.57 | 4.40 | 4.62 | 4.51 | 5.17 | 5.22 |
| *SD* | 0.97 | 0.87 | 0.87 | 0.88 | 0.79 | 1.01 | 0.80 | 0.72 |

*Note.* M_SC = math self-concept; G_SC = German self-concept; E_SC = English self-concept; P_SC = physics self-concept; B_SC = biology self-concept; FL_SC = second foreign language self-concept; VA_SC = visual arts self-concept; Mu_SC = music self-concept; M_IV = math intrinsic value; G_IV = German intrinsic value; E_IV = English intrinsic value; P_IV = physics intrinsic value; B_IV = biology intrinsic value; FL_IV = foreign language intrinsic value; VA_IV = visual arts intrinsic value; MU_IV = music intrinsic value; M_Ach = math achievement; G_Ach = German achievement; E_Ach = English achievement; P_Ach = physics achievement; B_Ach = biology achievement; FL_Ach = foreign language achievement; VA_Ach = visual arts achievement; Mu_Ach = music achievement.

Table S4

*Correlations among the academic achievement factors of the generalized internal/external frame of reference (GI/E) model (Model 2 of the main manuscript)*

|  | M_Ach | G_Ach | E_Ach | P_Ach | B_Ach | FL_Ach | VA_Ach |
| --- | --- | --- | --- | --- | --- | --- | --- |
| G_Ach | .509* |  |  |  |  |  |  |
| E_Ach | .432* | .602* |  |  |  |  |  |
| P_Ach | .613* | .493* | .437* |  |  |  |  |
| B_Ach | .432* | .452* | .401* | .491* |  |  |  |
| FL_Ach | .478* | .575* | .617* | .427* | .494* |  |  |
| VA_Ach | .184* | .360* | .276* | .248* | .337* | .238* |  |
| Mu_Ach | .323* | .419* | .347* | .297* | .347* | .376* | .356* |

*Note.* M_Ach = math achievement; G_Ach = German achievement; E_Ach = English achievement; P_Ach = physics achievement; B_Ach = biology achievement; FL_Ach = foreign language achievement; VA_Ach = visual arts achievement; Mu_Ach = music achievement.

* *p* < .05.

Table S5

*Correlations among academic self-concept and intrinsic value factors of the generalized internal/external frame of reference (GI/E) model (Model 2 of the main manuscript)*

|  | M_SC | G_SC | E_SC | P_SC | B_SC | FL_SC | VA_SC | Mu_SC | M_IV | G_IV | E_IV | P_IV | B_IV | FL_IV | VA_IV |
| --- | --- | --- | --- | --- | --- | --- | --- | --- | --- | --- | --- | --- | --- | --- | --- |
| G_SC | .062 |  |  |  |  |  |  |  |  |  |  |  |  |  |  |
| E_SC | .158* | .218* |  |  |  |  |  |  |  |  |  |  |  |  |  |
| P_SC | .439* | .100 | .233* |  |  |  |  |  |  |  |  |  |  |  |  |
| B_SC | .081 | .332* | .154* | .272* |  |  |  |  |  |  |  |  |  |  |  |
| FL_SC | .201* | .224* | .159* | .332* | .221* |  |  |  |  |  |  |  |  |  |  |
| VA_SC | -.033 | .189* | .181* | .067 | .233* | .091 |  |  |  |  |  |  |  |  |  |
| Mu_SC | .146 | .278* | .166* | .087 | .089 | .075 | .305* |  |  |  |  |  |  |  |  |
| M_IV | .725* | .101 | .118* | .347* | .013 | .175* | -.008 | .191* |  |  |  |  |  |  |  |
| G_IV | .013 | .634* | .105* | .120 | .190* | .176* | .188* | .164* | .155* |  |  |  |  |  |  |
| E_IV | .135* | .223* | .694* | .216* | .090 | .169* | .218* | .219* | .224* | .270* |  |  |  |  |  |
| P_IV | .303* | .033 | .201* | .810* | .230* | .232* | .065 | .129* | .356* | .108* | .306* |  |  |  |  |
| B_IV | .020 | .141* | .087 | .205* | .748* | .172* | .133* | .076 | .147* | .276* | .199* | .299* |  |  |  |
| FL_IV | .200* | .063 | .113* | .207* | .152* | .781* | .078 | .042 | .257* | .236* | .173* | .270* | .252* |  |  |
| VA_IV | -.023 | .100 | .103 | .100 | .119 | .059 | .779* | .208* | -.050 | .151* | .181* | .102 | .092 | .105 |  |
| Mu_IV | .114* | .131* | -.002 | .102 | -.006 | .047 | .154* | .724* | .144* | .185* | .143* | .249* | .054 | .127 | .263* |

*Note.* M_SC = math self-concept; G_SC = German self-concept; E_SC = English self-concept; P_SC = physics self-concept; B_SC = biology self-concept; FL_SC = second foreign language self-concept; VA_SC = visual arts self-concept; Mu_SC = music self-concept; M_IV = math intrinsic value; G_IV = German intrinsic value; E_IV = English intrinsic value; P_IV = physics intrinsic value; B_IV = biology intrinsic value; FL_IV = foreign language intrinsic value; VA_IV = visual arts intrinsic value; MU_IV = music intrinsic value.

* *p* < .05.

Table S6

*Standardized path coefficients of the mediated generalized internal/external frame of reference (GI/E) model (Model 3 of the main manuscript)*

|  | M_SC | G_SC | E_SC | P_SC | B_SC | FL_SC | VA_SC | Mu_SC | M_IV | G_IV | E_IV | P_IV | B_IV | FL_IV | VA_IV | Mu_IV |
| --- | --- | --- | --- | --- | --- | --- | --- | --- | --- | --- | --- | --- | --- | --- | --- | --- |
| M_Ach on | .760* | -.117* | -.124* | .227* | -.050 | .062 | -.116 | .061 | -.130 | -.066 | .027 | .013 | .025 | -.006 | .030 | .013 |
| G_Ach on | -.152* | .702* | .012 | -.207* | .031 | -.075 | -.003 | -.008 | .085 | -.021 | -.073 | -.005 | -.074 | .044 | .005 | .010 |
| E_Ach on | -.137* | .032 | .766* | -.027 | -.057 | .107 | -.058 | .050 | -.015 | -.029 | .161* | -.034 | -.026 | .002 | -.037 | -.071 |
| P_Ach on | .058 | -.096 | -.097 | .547* | .075 | -.158* | -.025 | -.114* | -.013 | -.042 | -.050 | -.027 | .063 | -.030 | -.035 | -.009 |
| B_Ach on | .041 | .020 | -.055 | .062 | .460* | -.132* | -.056 | -.030 | -.080 | .054 | -.013 | -.025 | -.081 | .028 | -.014 | -.005 |
| FL_Ach on | .045 | -.014 | -.073 | -.079 | .004 | .694* | .009 | .062 | .000 | .007 | -.051 | -.006 | .017 | -.138* | -.037 | -.032 |
| VA_Ach on | -.089 | .001 | -.083 | -.034 | .016 | .037 | .616* | -.030 | .010 | .111* | .040 | .042 | .070 | .059 | .050 | .029 |
| Mu_Ach on | -.019 | .025 | .089* | -.038 | .060 | .062 | .044 | .537* | .016 | .024 | -.052 | -.061 | -.085 | -.031 | .021 | -.013 |
| M_SC on |  |  |  |  |  |  |  |  | .887* |  |  |  |  |  |  |  |
| D _SC on |  |  |  |  |  |  |  |  |  | .722* |  |  |  |  |  |  |
| E_SC on |  |  |  |  |  |  |  |  |  |  | .743* |  |  |  |  |  |
| P_SC on |  |  |  |  |  |  |  |  |  |  |  | .875* |  |  |  |  |
| B_SC on |  |  |  |  |  |  |  |  |  |  |  |  | .817* |  |  |  |
| FL_SC on |  |  |  |  |  |  |  |  |  |  |  |  |  | .905* |  |  |
| VA_SC on |  |  |  |  |  |  |  |  |  |  |  |  |  |  | .820* |  |
| Mu_SC on |  |  |  |  |  |  |  |  |  |  |  |  |  |  |  | .789* |

*Note.* M_SC = math self-concept; G_SC = German self-concept; E_SC = English self-concept; P_SC = physics self-concept; B_SC = biology self-concept; FL_SC = second foreign language self-concept; VA_SC = visual arts self-concept; Mu_SC = music self-concept; M_IV = math intrinsic value; G_IV = German intrinsic value; E_IV = English intrinsic value; P_IV = physics intrinsic value; B_IV = biology intrinsic value; FL_IV = foreign language intrinsic value; VA_IV = visual arts intrinsic value; MU_IV = music intrinsic value.

* *p* < .05.

Table S7

*Correlations among the achievement factors of the mediated generalized internal/external frame of reference (GI/E) model (Model 3 of the main manuscript)*

|  | M_Ach | G_Ach | E_Ach | P_Ach | B_Ach | FL_Ach | VA_Ach |
| --- | --- | --- | --- | --- | --- | --- | --- |
| G_Ach | .510* |  |  |  |  |  |  |
| E_Ach | .433* | .602* |  |  |  |  |  |
| P_Ach | .614* | .494* | .439* |  |  |  |  |
| B_Ach | .433* | .453* | .403* | .492* |  |  |  |
| FL_Ach | .479* | .577* | .618* | .429* | .495* |  |  |
| VA_Ach | .184* | .360* | .277* | .249* | .337* | .239* |  |
| Mu_ Ach | .324* | .420* | .348* | .299* | .348* | .376* | .357* |

*Note.* M_Ach = math achievement; G_Ach = German achievement; E_Ach = English achievement; P_Ach = physics achievement; B_Ach = biology achievement; FL_Ach = foreign language achievement; VA_Ach = visual arts achievement; Mu_Ach = music achievement.

* *p* < .05.

Table S8

*Correlations among the intrinsic value factors of the mediated generalized internal/external frame of reference (GI/E) model (Model 3 of the main manuscript)*

|  | M_IV | G_IV | E_IV | P_IV | B_IV | FL_IV | VA_IV |
| --- | --- | --- | --- | --- | --- | --- | --- |
| G_IV | .215* |  |  |  |  |  |  |
| E_IV | .266* | .287* |  |  |  |  |  |
| P_IV | .281* | .092 | .314* |  |  |  |  |
| B_IV | .353* | .367* | .314* | .331* |  |  |  |
| FL_IV | .218* | .334* | .125 | .337* | .320* |  |  |
| VA_IV | -.099 | .076 | .092 | .052 | .065 | .135 |  |
| Mu_IV | .020 | .195* | .134 | .334* | .107 | .227* | .358* |

*Note.* M_IV = math intrinsic value; G_IV = German intrinsic value; E_IV = English intrinsic value; P_IV = physics intrinsic value; B_IV = biology intrinsic value; FL_IV = foreign language intrinsic value; VA_IV = visual arts intrinsic value; MU_IV = music intrinsic value.

* *p* < .05.

*
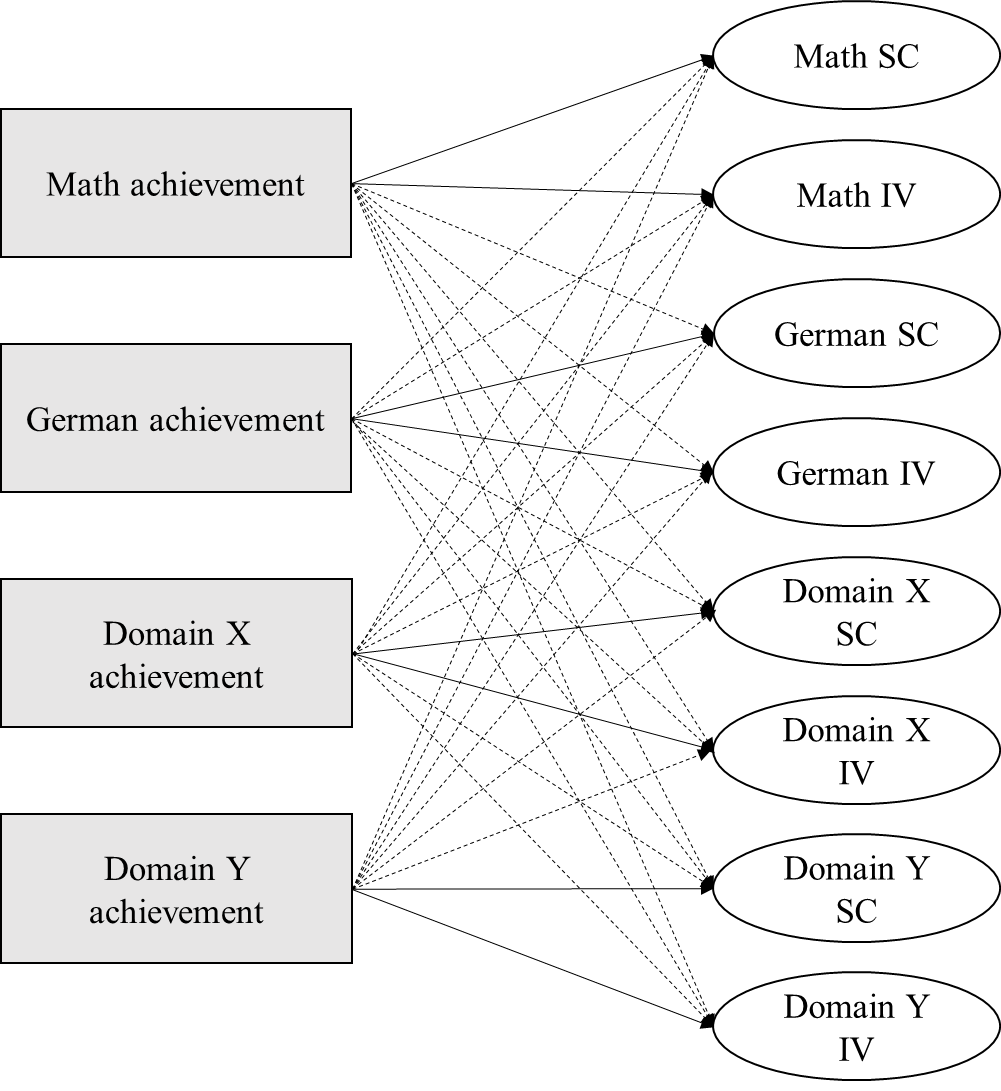
*

*Figure S1*.

An exemplary generalized intrinsic/extrinsic frame of reference model

*Note.* SC = self-concept, IV = intrinsic value. The solid paths represent within-domain relations depicting social comparisons; the dashed paths represent cross-domain relations depicting dimensional comparisons. The correlations among achievement factors and the correlations among self-concept factors, among intrinsic value factors, and between self-concept and intrinsic value factors are not depicted for the sake of readability.

*Figure S2*.

An exemplary mediated generalized intrinsic/extrinsic frame of reference model

*Note.* SC = self-concept, IV = intrinsic value. The solid paths represent within-domain relations depicting social comparisons; the dashed paths represent cross-domain relations depicting dimensional comparisons. The correlations among achievement factors and the correlations among self-concept factors, among intrinsic value factors, and between self-concept and intrinsic value factors are not depicted for the sake of readability.
